# Editorial: Induced Pluripotent Stem Cell-Based Disease Modeling and Drug Discovery: Can We Recapitulate Cardiovascular Disease on a Culture Dish?

**DOI:** 10.3389/fcell.2021.831304

**Published:** 2022-01-31

**Authors:** Shinsuke Yuasa, Masayuki Yazawa, Jong-Kook Lee

**Affiliations:** ^1^ Department of Cardiology, Keio University School of Medicine, Tokyo, Japan; ^2^ Department of Rehabilitation and Regenerative Medicine, Vagelos College of Physicians and Surgeons, Columbia University, New York City, NY, United States; ^3^ Department of Molecular Pharmacology and Therapeutics, Vagelos College of Physicians and Surgeons, Columbia University, New York City, NY, United States; ^4^ Columbia Stem Cell Initiative, Vagelos College of Physicians and Surgeons, Columbia University, New York City, NY, United States; ^5^ Department of Cardiovascular Regenerative Medicine, Osaka University Graduate School of Medicine, Suita, Japan

**Keywords:** iPS cell, cardiomyocyte, endothelial cell, disease modeling, drug discovery

Since induced pluripotent stem (iPS) cells were first generated in 2006 (Takahashi and Yamanaka, 2006), human iPS cell culture and differentiation into various cell types have been widely established as research platforms for elucidating disease mechanisms and for drug discovery research, and promising cell sources for regenerative medicine (Tanaka et al., 2015). Human iPS cells can be generated from patient’s somatic cells, such as skin fibroblasts or blood cells, and the approach using iPS cells offers an ethical advantage over embryonic stem cells (Seki et al., 2010; Seki et al., 2012). There have been a variety of established methods to differentiate many cell types from human iPS cells. In terms of cardiovascular disease, recent accumulating progress has enabled human iPS cells to be efficiently differentiated and purified into subtypes of cardiomyocytes, such as ventricular-, atrial- and nodal-like myocytes, as well as smooth muscle cells and endothelial cells. Based on these advances, disease-specific iPS cells can be generated from patients with intractable cardiovascular diseases, such as inherited cardiomyopathies and arrhythmic disorders. There are substantial reports to elucidate the molecular and cellular mechanisms underlying disease onset and/or the pathophysiological process through various disease-specific iPS cell studies (Yazawa et al., 2011; Egashira et al., 2012; Tanaka et al., 2014; Yasutake et al., 2021). It is also conceivable that iPS cell-derived cardiomyocytes can be utilized for drug screening. For robust applications using human iPS cells for research and clinics, there remains several barriers to be overcome, such as immaturity of iPS cell-derived cardiomyocytes, lack of cell-cell interaction, and absence of organ structure. Many studies still struggle against these issues, which should be solved step by step.

Although iPS cells are supposedly differentiated into any types of cells, most of the differentiated cells that are currently available show immature/fetal feature. To model adult-onset diseases, it is required to mature the differentiated cells. In terms of regeneration therapy, immature cardiomyocyte transplantation may induce arrhythmic event and reduce the efficacy of functional recovery. To mature cardiomyocytes, it is important to understand the difference between embryonic and adult cardiomyocytes. Anzai et al. identified gene sets to monitor the developmental stage in murine and human cardiomyocytes. Comparative transcriptome data analysis of mouse and human hearts in several developmental stages revealed the difference and similarity of two species. While some reports previously showed maturation markers, the proposed markers were either mouse specific or human specific. Importantly, identified gene sets can be markers for both mouse and human cardiomyocyte maturation. Li et al. reported their cardiomyocyte maturation method in human iPS cell-derived cardiomyocytes. Aligned fiber substrate culture induced mature-like properties including rod shape morphology, shortened action potential duration, accelerated conduction velocity, and elevated adult-type gene expression. Contractility of the heart increases as its beating rate is elevated, which is observed in human matured heart, but not in mouse heart and immature iPS cell-derived cardiomyocytes. Izumi-Nakaseko et al. reported that motion directional regulation by electrical pacing could induce positive force-frequency relationship in monolayers of human iPS cell-derived cardiomyocytes. These findings facilitate us to establish sophisticated methods to mature cardiomyocytes and analysis method to understand the molecular and cellular pathophysiological mechanisms underlying heart diseases.

To model human cardiovascular disease and establish regeneration therapy using iPS cell-derived cells, it is essential for us to develop new methods to differentiate iPS cells into cardiovascular cells and understand the properties of iPS cell-derived cells. Although endothelial progenitor cells (EPCs) have been used for vascular regeneration therapy, it is inefficient to obtain EPCs from adult donors. Farkas et al. reported the efficient protocol to differentiate EPCs from human iPS cells. As for EPCs, immature phenotype is not disadvantageous because mature endothelial cells lack angiogenic and vasculogenic potential. However, to use iPS cell-derived immature cardiomyocytes, it is critically important to understand the difference from adult cardiomyocytes. Muscular dystrophies are caused by the mutation in genes encoding the protein involved in the dystrophin-associated protein complex (DAPC). Cardiomyopathies are crucial phenotypes and could be a lethal cause. To model this disease, it is important to examine the expression and function of DAPC in iPS cell-derived cardiomyocytes. Gilbert et al. showed the robust expression of DYSTROPHIN, but the absence of several other DAPC proteins, suggesting that it is still difficult to model the disease using current immature cardiomyocytes. The type 2 ryanodine receptor (RYR2) is an essential Ca^2+^ release channel of sarcoplasmic reticulum (SR) in adult cardiomyocyte. Luo et al., generated *RYR2*−/− iPS cell line and investigated the role of RYR2 in iPS cell-derived cardiomyocytes. RYR2 is not required for cardiomyocyte differentiation but plays a role in survival and contractile function. Without RyR2, another calcium release channel, IP3R mediates Ca^2+^ release as a compensatory mechanism for Ca^2+^ handling in iPS cell-derived cardiomyocyte.

Atrial fibrillation (AF) is the most common cardiac arrhythmic disease. Loss of function and gain of function mutations in *KCNA5*, encoding the Kv1.5α-subunit of the ion channel carrying the atrial-specific ultrarapid delayed rectifier K^+^ current (I_kur_) are reported in approximately 10% of the patients with AF. Hilderink et al. examined the effects of virtual I_Kur_ injection in iPS cell-derived atrial-like cardiomyocyte because native I_Kur_ density is too small to be examined. Virtual modulation study revealed that a decrease in *I*_Kur_, mimicking loss-of-function mutations, significantly prolonged action potential duration, but an increase in *I*_Kur_, mimicking gain-of-function mutations, mildly shortened that in iPS cell-derived atrial-like cardiomyocyte. Long QT (LQT) syndrome is an inherited life-threatening arrhythmogenic disease. Although recent report showed the efficacy of β-blockers on the LQT type3 (LQT3) (Wilde et al., 2016), the pharmacological mechanism remains unclear. Hirose et al. modeled the LQT3 and confirmed the efficacy of β-blocker in the model. Interestingly, β-blocker reduced the disease-causing late sodium current in the presence of guanosine diphosphate βs (GDPβs), an inhibitor of G proteins, suggesting that the effect of β-blocker may be independent of β-adrenergic receptor. Hypoplastic left heart syndrome (HLHS) is a severe form of congenital heart disease. While genetic variants in *MYH6* are reported, the disease mechanism remains unknown. Kim et al. model HLHS by iPS cell-derived cardiomyocytes and showed multiple impairment in cardiomyocyte differentiation, sarcomere organization, slower contraction, and decreased velocity phenotypes. HLHS patients sample showed sarcomere disorganization in atrial but not ventricular tissues. These results suggest that reduced contractility in atrium of HLHS patients may negatively affect hemodynamics and result in the development of a left ventricle.

Cardiovascular diseases are highly prevalent globally as a main cause of death. Several therapeutic strategies have been developed, such as medical therapy, catheter intervention, cardiac assist device, and heart transplantation. However, the therapeutics remain insufficient, and we still require innovative approaches to improve the therapeutics. To develop the therapeutics further, it is important to understand the disease pathogenesis and develop the drug discovery and optimization platforms. Various innovative analysis methods have been proposed, developed, and contributed to the cardiovascular research (Kusumoto et al., 2018; Kusumoto and Yuasa, 2019; Kusumoto et al., 2021). Therefore, the research using human iPS cells with genetic variants is still highly valuable. Continuous iPS cell study will allow us to understand the diseases and develop the innovative therapy to improve human health.
